# Data on safe hydrogen production from the solar photovoltaic solar panel through alkaline electrolyser under Algerian climate

**DOI:** 10.1016/j.dib.2018.10.106

**Published:** 2018-10-27

**Authors:** Chawki Ameur Menad, Rabah Gomri, Mohamed Bouchahdane

**Affiliations:** a Climatic Department, University Mentouri Constantine 1, 25000 Constantine, Algeria; b Department of Power and Control Institute of Electrical and Electronic Engineering, IGEE, Boumerdes, Algeria

## Abstract

This data article is about hydrogen production from the solar photovoltaic solar panel through alkaline electrolyser under Algerian climate with the application of safe technology. Several pathways have been given different technologies for hydrogen production which were based on the experimental scale, where the catalysis of water by using alkaline electrolyser has taken the major part of the collecting data. However, in this data article, the photovoltaic solar system is integrated with a controller, an ammeter, a voltmeter, and an alkaline type water electrolyser (the electrolyte used is Na–OH). In fact, the alkaline electrolyser is the most common technology for the production of hydrogen through solar energy. Many technical problems have been involved to reduce the energy efficiency of the new design of the solar system, especially in photovoltaic panel solar system. One of the proposed solutions in this data is integrating of a numerical relay REF542 plus with a solar photovoltaic power station in the city of Adrar, which is located in the south of Algeria, to protect the electrical grid from over-current. This data is related to following references (Tributsch, 2008; Kai and Zhang, 2010; Chennouf et al., 2013).

**Specifications table**

**Table:** 

Subject area	Physics,
More specific subject area	Safety of Photovoltaic Panel for hydrogen production.
Type of data	Table, text file, figure
How data was acquired	CMC365
Data format	Filtered, analyzed
Experimental factors	Hydrogen production from photovoltaic solar system is integrated with a controller, an ammeter, a voltmeter, and an alkaline type water electrolyser (the electrolyte used is Na–OH). Moreover; safety of solar system has been taken in consideration through connecting CMC356 with 220 V AC, with the relay REF542plus
Experimental features	The experience is based on linking a numerical relay including the CMC356 with photovoltaic panels in city of Adrar. Moreover the photovoltaic panels are connected to alkaline electrolyser for hydrogen production from catalysis of water.
Data source location	City of Adrar, Algeria
Data accessibility	Data is available with this article
Related research article	[3] H. Tributsch, Photovoltaic hydrogen generation. Int. J. Hydrogen Energy, 33(2008), pp. 5911–5930.
	[4] Z. Kai, D. Zhang, Recent progress in alkaline water electrolysis for hydrogen production and applications. Progress in Energy and Combustion Science, 36(2010), pp. 307–326.
	[5] N. Chennouf, N. Settou, B. Negrou, K. Bouziane, B. Dokkar, Etude d’une Installation de production d’hydrogène solaire par électrolyse de l’eau dans la région d’Ouargla. Annales des Sciences et Technologie, 5(2013).

**Value of the data**

• Data can be used in exploiting the alkaline electrolyzer for the production of hydrogen through photovoltaic solar energy under Algerian climate.
• Data can be used in combination of a numerical relay REF542 plus with a solar photovoltaic power station for securing the process of hydrogen production.
• Data can be used for understanding the suitable region for hydrogen production from solar energy under Algerian climate.
• Data can be used in combination the a numerical relay REF542 plus with the CMC356 and photovoltaic solar panels in the city of Adrar which is considered one of the best regions for solar energy in Algeria.

## 1 Data

This data article describes the essential steps of hydrogen production from the photovoltaic solar panel through alkaline electrolyser under Algerian climate in safety technology. The available data of the selected photovoltaic solar panel are based on the following characteristics (Table 1, Table 2, Table 3).

**Table 1:** Electrical specifications of the photovoltaic panel.

	Unit	Module	Module	Module
Nominal power	W	190	195	200
Open circuit voltage	V	44.5	44.5	44.5
Short circuit current	A	5.52	5.77	5.92
Voltage Vmpp	V	36.5	36.5	36.5
Current mpp Impp	I	5.21	5.34	5.48
Maximum voltage system MVS	V	1000	1000	1000
Maximum load of fuses (A)	A	10	10	10

**Table 2:** Temperature factor (Cell) of the photovoltaic panel.

Nominal operating temperature	NOCT	(45+2 °C)
		(45−2 °C)
Power temperature coefficient	%/°C	−0.37
Current temperature coefficient	%/°C	+0.033
Voltage temperature coefficient	%/°C	−0.241

**Table 3:** Mechanical characteristics of the photovoltaic panel.

Cell type	Mono-crystalline cell with anti-reflection
Number of cells per module	72 cells (61 × 2)
Dimensions of the cell	125 × 125 mm
Module dimensions	1580 × 808 × 45mm
Module weight	14.5 kg
Frame	Anodized aluminum alloy
Type of glass	Heat-tempered glass 3.2 mm thick
Junction box and connector	IP65 by-pass diodes and cables compatible with MC4 connector
Operating temperature	−40°C to 85 °C

STC: 1000 W/m^2^, AM1.5, and 25 °C cell temperature; NOCT, nominal operating cell temperature.

**Fig. 1:**
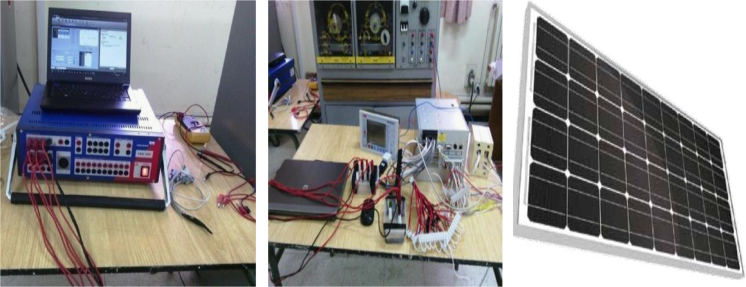
REF452 plus and CMC356 connected together with the photovoltaic solar panel.

In this data, the photovoltaic solar system is integrated with a controller, an ammeter, a voltmeter, and an alkaline type water electrolyser (the electrolyte used is Na–OH). The experience is based on linking a numerical relay including the CMC356 with photovoltaic solar panels, and test equipment associated with Test Universe software. The communication between CMC356 and the PC where the Test Universe is installed is realised through an Ethernet cable. After finishing the connections above, the CMC356 and the relay were turned on; the relay is ready to be tested. Fig. 1 shows the process of hydrogen production through solar photovoltaic panel.

## 2 Understanding of the solar panel and electrolyser testing

This data aims to develop an installation of solar hydrogen production where the photovoltaic solar system is integrated with a controller, an ammeter, a voltmeter, and an alkaline type water electrolyser (the electrolyte used is Na–OH). The energy produced by photovoltaic panel is absorbed by the alkaline electrolyser to produce hydrogen and oxygen through electrolysis of water. Each step of the process will be conducted at the laboratory scale. Several pathways have been developed for hydrogen production based on the experimental scale, where the catalysis of water by using alkaline electrolyser has taken the major part of the collecting data. The purpose of this paper is obtaining hydrogen from clean source of renewable energy which is solar energy. This technology is currently one of the most important solutions for chemical and petrochemical industries. Other data describes a part of hydrogen production from solar energy were developed. One of available data is based on solar energy due to its availability in Algeria without risk known by fossil energy sources [1]. Moreover, combination of solar energy with alkaline electrolyser is considered the most profitable and most protective method for the environment [2].

### 2.1 Principle of energy conversion

The equivalent electrical diagram in a photovoltaic solar cell is shown in Fig. 1. This circuit comprises a current source and a diode connected in parallel position. The current source supplies a current *I*_ph_, directly proportional to the intensity of light, where the diode represents the p–n junction of the solar cell.

The electric power output of the PV system is:

(1) $$P_{\mathrm{el}}=I\cdot V$$

Maximum power is [3]:

(2) $$P_{\max}=\left(I\cdot V\right)\max=\mathrm{VOC}\cdot \mathrm{ICC}\cdot \mathrm{FF}$$

The yield of the PV system, *η* [3]:

(3) $$\eta =\frac{V_{\mathrm{mp}}I_{\mathrm{mp}}}{P\cdot C}=\frac{V_{\mathrm{oc}}\cdot \mathrm{ICC}\cdot \mathrm{FF}}{\mathrm{PC}}$$

**Fig. 2:**
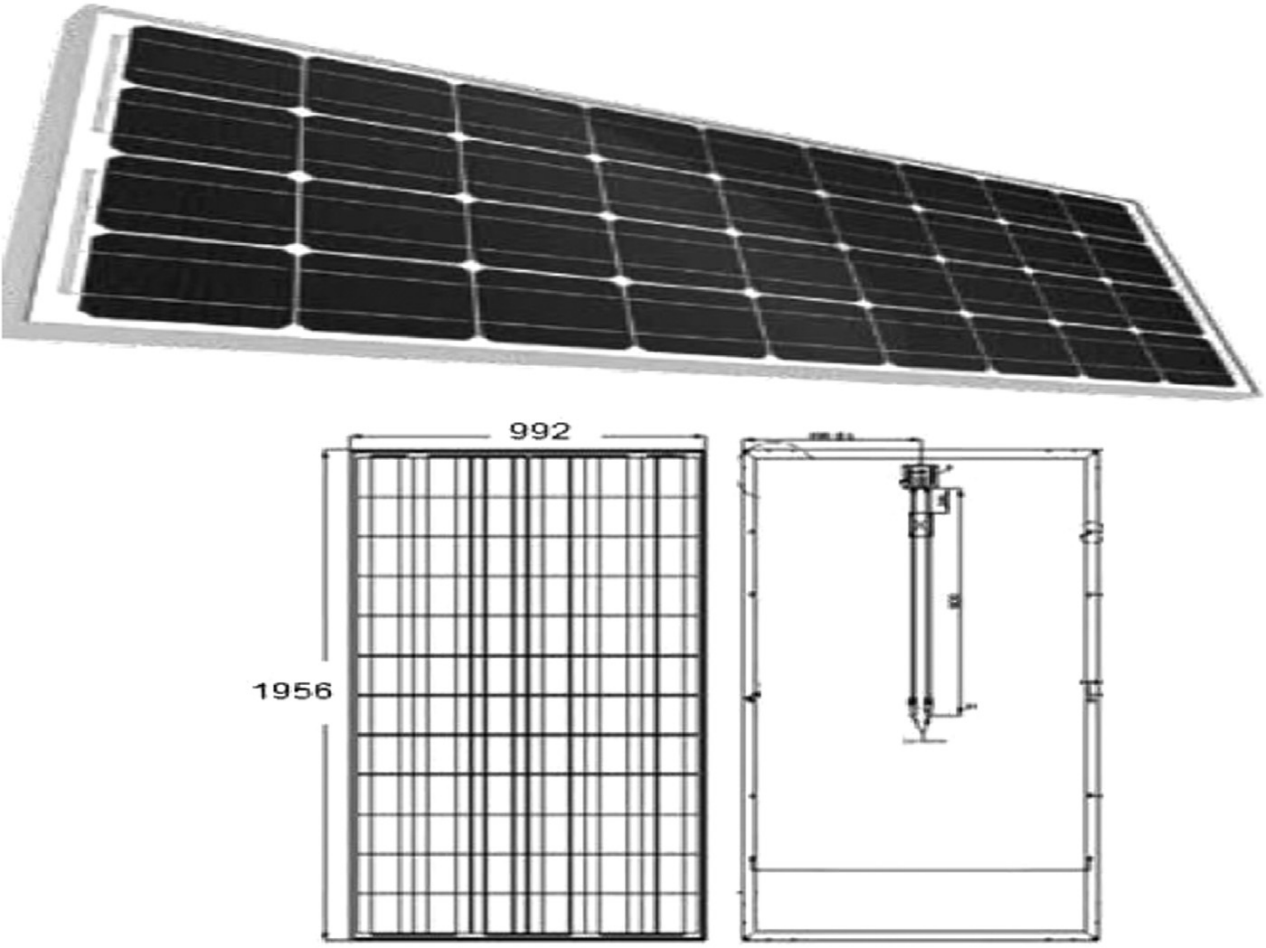
Photovoltaic solar panel.

Fig. 2 shows the selected photovoltaic solar panel.

### 2.2 Alkaline electrolyser principle

Alkaline electrolyser is the most common technology for the production of hydrogen through solar energy, the electrolyte is based on (Na–OH). The anodic and cathodic reactions are described below [4].

(4) $$\mathrm{At}\;\mathrm{the}\;\mathrm{Anode}:2{\mathrm{OH}}^{-}\to 0.5O_{2}+H_{2}O+2e^{-}$$

(5) $$\mathrm{At}\;\mathrm{the}\;\mathrm{Cathode}:2H_{2}O+2e^{-}\to H_{2}+2{\mathrm{OH}}^{-}$$

### 2.3 Calculation parameters

These calculations are based on available described data about understanding of the solar panel and electrolyser testing for hydrogen production through alkaline electrolyser [5].

Volume flow rate of hydrogen:

(6) $$Q_{a}=\frac{V_{H}}{t}$$

Power absorbed by the electrolysis:

(7) $$P_{a}=V\cdot I$$

Power output by electrolysis:

(8) $$P_{u}=\mathrm{PCI}\cdot Q\cdot \rho$$

The electrical energy consumed

(9) $$W=P_{a}\cdot t$$

Useful efficiency:

(10) $$R_{u}=\mathrm{PCI}\cdot \frac{V_{H}}{P_{a}t}\cdot \rho$$

Solar yield:

(11) $$R_{s}=\mathrm{PCI}\cdot \frac{V_{H}}{P_{g}t}\cdot \mathrm{0\rho}$$

### 2.4 Hydrogen production

Hydrogen production from renewable energy resources has taken an important debate in the last decades where the researchers have been developed many projects. A lot of research papers have been developed to understand the combination between hydrogen production and solar energy resources. Ghribi et al. [6] proposed a mathematical model of hydrogen production system using the electrolyser to maximize hydrogen production. The obtained results have explained that with the Fresnel reflector, the H_2_ produced is about 0.14 kgH_2_/m^2^/day. However, this value increased to 0.19 kgH_2_/m^2^/day for a suitable period [6].

### 2.5 Efficiency

Alkaline electrolyser is one of the positive pathways for hydrogen production, with real challenges which are based on cost and maintenance to increase energy efficiency, reducing energy consumption, safety, durability, and reliability [7], [8]. In this data, an electrolyser with power capacity of 52.5 kWh/kg is considered [9]. The computation of the hydrogen mass produced from geothermal energy is described as follows:

(12) $$M_{H2}=\frac{E_{\mathrm{EL}}}{{\mathrm{LHV}}_{H2}}=\frac{\eta_{\mathrm{EL}}E_{\mathrm{EGS}}}{{\mathrm{LHV}}_{H2}}$$

where, EEL is the energy required for electrolyser and hEL is the efficiency of the electrolyser. The lower heating value LHV of the produced hydrogen is taken as 33.31 kWh/kg [9].

### 2.6 Electrochemical process

**Fig. 3:**
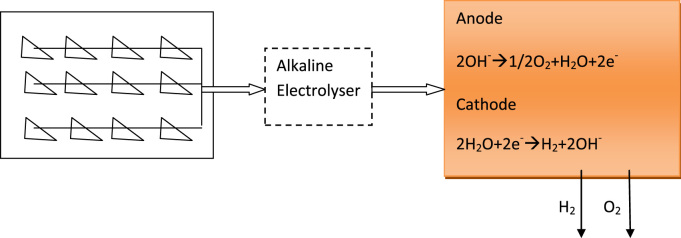
Process of hydrogen production.

Producing hydrogen from photovoltaic solar energy in Algeria is the main purpose of the proposed model. Fig. 3 shows two different parts: The thermal solar part, and hydrogen production cycle part.

• In the solar field, the photovoltaic solar panels produce electricity for generating the alkaline electrolyser.

• The second part proofed that the proposed model mentions that there is one pathway for producing hydrogen through catalysis of water.

### 2.7 Photovoltaic solar field

The process of hydrogen production is shown in Fig. 3.

## 3 Description of photovoltaic solar panel

Many research articles have published to explain the goal of hydrogen production from solar photovoltaic systems. Becherif et al. [10] have described Hydrogen production horizon using solar energy photovoltaic panels in Biskra, which is located in Algeria. Moreover, the experience is devoted to understand two different models for the areas solar radiation, where the solar radiation model on a horizontal and on a tilted and oriented PV panel is described.

Kirati et al. [11] have discussed the power of hybrid energy system based on photovoltaic panels for hydrogen production in Adrar region (Algeria) according to operating parameters, climatic conditions, and the load of the site of Adrar. The cell electrolyzer model permits to predict the production rate of hydrogen with accuracy.

In this part a photovoltaic panel has been installed in city of Adrar (Algeria) to produce electricity for hydrogen production.

• The efficiency of the studied case is given by:

(13) $$\eta =\frac{P_{\mathrm{solar}\;\mathrm{panel}}}{P_{\mathrm{sun}}}$$

$P_{\mathrm{solar}\;\mathrm{panel}}$ The power produced by the solar panel.
$P_{\mathrm{sun}}$ The solar irradiation absorbed by the sun.

Shockley and Queisser [12] and Tiedje et al. [13] have proposed a different method for calculating the maximum efficiency of the photovoltaic solar panels. However, the available data used photovoltaic solar panel to describe transformation sun irradiation to electricity. The following equation explains the amount of electricity produced by the solar panel.

(14) $$P_{\mathrm{solar}\;\mathrm{panel}}=q\cdot (\varphi_{1}-\varphi_{2})\mu$$

where $q.(\varphi_{1}-\varphi_{2})\mu$ is the absorbed energy by the solar panel based on the quasi-fermi level separation in the maximum case.Where $\varphi_{1}$ is the absorbed flux and $\varphi_{2}$ is the emission flux. The Plank׳s equation is unique solution for calculating of the flux for different configurations which are the total absorbed flux and the emission flux.

(15) $$\varphi (E_{a},E_{b},\mu ,T)=\mathrm{Cf}\frac{2\cdot \pi }{h^{3}C^{2}}\int_{E_{a}}^{E_{b}}\frac{E^{2}}{e^{(\frac{E-\mu }{\mathrm{kT}})}-1}\mathrm{dE}$$

In case of absorbed flux: μ=0

For emission flux: μǂ0 (different of zero).

## 4 Alkaline electrolyser for hydrogen production

In this part, alkaline electrolyser has been used to produce hydrogen from water electrolysis (Eqs. (4), (5)).

An alkaline electrolyser has been selected in this description data. The hydrogen produced with high purity can then be used to cover the energy demands for the future generations. In this analysis, an alkaline electrolyser with power capacity of 52.5 kWh/kg is considered (which is equivalent to about 75% in efficiency).

## 5 Experimental design, materials, and methods

The design of this data has been divided into two parts which are:

• Explaining the safety of solar photovoltaic panels for producing electricity. This part shows the process of over-current protection by installing the necessary equipments in photovoltaic solar power station.
• Hydrogen production by alkaline electrolyser from the electricity produced by the photovoltaic panel through electrolysis of water.

### 5.1 Safety of the photovoltaic solar panel

The safety of photovoltaic solar panel is based on understanding the over-current protection. Extensive researches have been published, especially to describe over-current protection for the safety of solar photovoltaic systems. Sherbilla et al. [14] have presented a practical and versatile setting profile for over-current protective elements, and description of connection and distribution generators (DGs), which are commonly utilized with renewable energy resources including solar photovoltaic. Costa et al. [15] have proposed new way which is based on the boundary discrete wavelet transform for over-current protection by using conventional and modern protection trends. However, a distribution system with DG was modeled in a real-time digital simulator. Mahat et al. [16] have published an important data about a Simple Adaptive Over-current protection of distribution systems with distributed generation to explain the aim of using over-current protection in an islanded distribution system. Haron et al. [17] have discussed analysis, solutions of over-current protection issues in a microgrid, by using relay over-reaching, and relay under-reaching to resolve the issue of over-current protection in the radial distribution network.

**Fig. 4:**
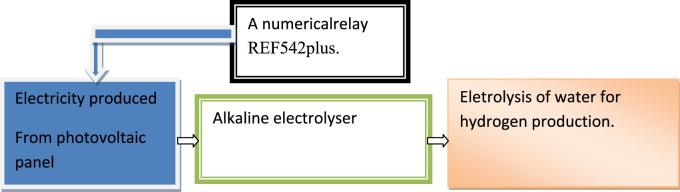
Process of hydrogen production (alkaline electrolyser).

Fig. 4 shows REF452 plus and CMC356 connected together in the laboratory for tests of safety.

### 5.2 Possibility of hydrogen production from solar photovoltaic solar panel using the safe technology relay REF542 plus

This data has given a strong proof that the hydrogen production from photovoltaic panel in city of Adrar, which is located in the south of Algeria. Both controlled and non-controlled over-current protection of the photovoltaic have an impact on quality and purity of hydrogen production under Algerian climate.

**Fig. 5:**
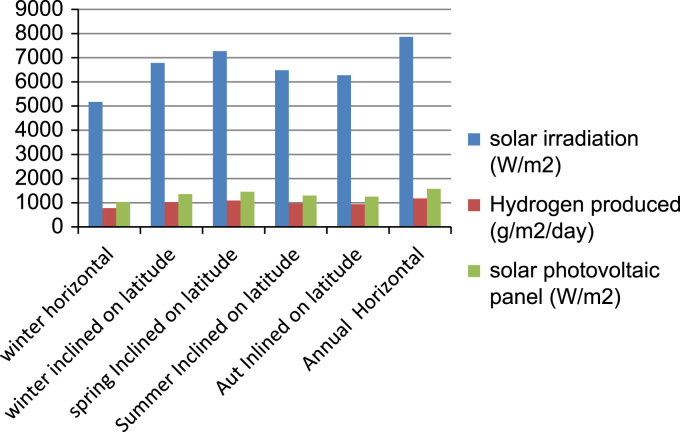
The impact of controlled over-current in photovoltaic solar panel on hydrogen production from alkaline electrolyser.

Fig. 5 shows the impact of controlled over-current in photovoltaic solar panel on hydrogen production from alkaline electrolyser. In this case the amount of hydrogen produced is varied between 0.7755 kgH_2_/m^2^/day and 1.179 kgH_2_/m^2^/day. Solar irradiation in city of Adrar is considered a suitable region for hydrogen production through alkaline electrolyser from photovoltaic panel with controlled over-current.

**Fig. 6:**
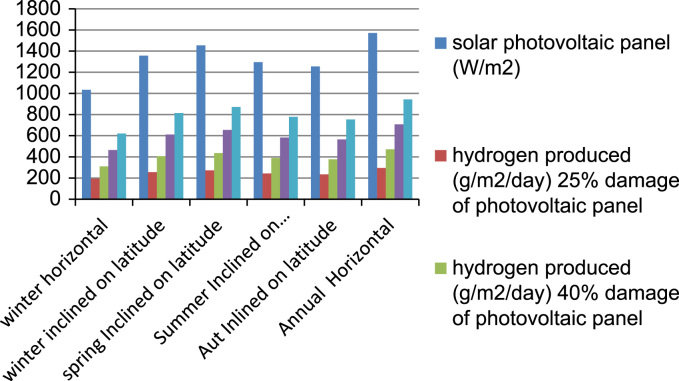
Impact of over-current non-controlled in photovoltaic solar panel on hydrogen production from alkaline electrolyser.

Fig. 6 shows the impact of non-controlled over-current in photovoltaic solar panel on hydrogen production from alkaline electrolyser. In this case the amount of hydrogen produced has been reduced, due to damage of solar cells in one side, and electrical faults in the grid between the solar power station and the electrolyser in the other side. Safety of photovoltaic solar power station is necessary for securing the continuation of hydrogen production by installing numerical relay.

Design and installing solar photovoltaic panels in the hard environment is based on the safety of the solar system from dust and wind. However, this data has mentioned another important factor which over-current protection for securing hydrogen production in a different critical situation such humidity. In fact, hydrogen production from electrolysis of water has taken a major part in last decades to cover the energy needs in Algeria, but this data is considered one of the basic solid foundation for the researcher to start developing new energetic design systems to improve energy efficiency. From the measurement of different climatic factors which are humidity, temperature, solar irradiation, and the available data, we are able to produce hydrogen and electricity in the same time from photovoltaic solar energy systems.
