# Supplementary material for: Data on safe hydrogen production from the solar photovoltaic solar panel through alkaline electrolyser under Algerian climate
Source: Data Brief. 2018 Oct 27;21:1051–60. doi: 10.1016/j.dib.2018.10.106 (PMC6226822; doi:10.1016/j.dib.2018.10.106)
Supplement: Supplementary file 1 — Supplementary material [file mmc1.pdf]

Chawki Ameur menad

PhD student at the University

Mentouri-Constantine Algeria

F2 51 lidderdale Road Liverpool

L15 3JG UK

02/06/2018

Dear Data in Brief

I Mr : Chawki Ameur menad confirm that this work is original and has not been published elsewhere, nor is it currently under consideration for publication elsewhere.

We have no conflicts of interest to disclose.

Kind regards

Chawki Ameur menad.
